# Cancer incidence and spectrum among children with genetically confirmed Beckwith-Wiedemann spectrum in Germany: a retrospective cohort study

**DOI:** 10.1038/s41416-020-0911-x

**Published:** 2020-05-26

**Authors:** Sümeyye Cöktü, Claudia Spix, Melanie Kaiser, Jasmin Beygo, Stephanie Kleinle, Nadine Bachmann, Nicolai Kohlschmidt, Dirk Prawitt, Alf Beckmann, Ruediger Klaes, Claudia Nevinny-Stickel-Hinzpeter, Steffi Döhnert, Cornelia Kraus, Gundula Kadgien, Inga Vater, Saskia Biskup, Michael Kutsche, Jürgen Kohlhase, Thomas Eggermann, Martin Zenker, Christian P. Kratz

**Affiliations:** 1 Department of Pediatric Hematology and Oncology, Hannover Medical School, Carl-Neuberg-Str. 1, Hannover, 30625 Germany; 2 German Childhood Cancer Registry, Institute for Medical Biostatistics, Epidemiology and Informatics, University Medical Center Mainz, Obere Zahlbacher Str. 69, Mainz, 55131 Germany; 3 Institut für Humangenetik, Universitätsklinikum Essen, Universität Duisburg-Essen, Hufelandstr. 55, Essen, 45147 Germany; 4 MGZ, Medizinisch Genetisches Zentrum München, Bayerstr. 3-5, München, 80335 Germany; 5 Bioscientia Ingelheim, Konrad-Adenauer-Str.17, Ingelheim, 55218 Germany; 6 Limbach Genetics, Medizinische Genetik Mainz Prof. Bergmann & Kollegen, Mainz, Germany; 7 Institut für Klinische Genetik und Tumorgenetik Bonn, Maximilianstraße 28d, Bonn, 53111 Germany; 8 Center for Paediatrics and Adolescent Medicine, University Medical Center, Langenbeckstr. 1, Mainz, 55101 Germany; 9 MVZ Dr. Eberhard & Partner Dortmund, Humangenetik, Brauhausstr.4, Dortmund, 44137 Germany; 10 SYNLAB MVZ Humangenetik Mannheim GmbH, Harrlachweg 1, Mannheim, 68163 Germany; 11 SYNLAB MVZ Humane Genetik München, Lindwurmstr. 23, München, 80337 Germany; 12 Mitteldeutscher Praxisverbund Humangenetik - Überörtliche Gemeinschaftspraxis, Friedrichstr. 38/40, Dresden, 01067 Germany; 13 Humangenetisches Institut, Universitätsklinikum Erlangen, Friedrich-Alexander-Universität Erlangen-Nürnberg (FAU), Schwabachanlage 10, Erlangen, 91054 Germany; 14 MVZ für Humangenetik und Molekularpathologie, Robert-Koch-Str. 10, Rostock, 18059 Germany; 15 Institut für Humangenetik, Universitätsklinikum Schleswig-Holstein, Campus Kiel, Arnold-Heller-Str. 3, Haus 10, Kiel, 24105 Germany; 16 Praxis für Humangenetik / CeGaT, Paul-Ehrlich-Str. 23, Tübingen, 72076 Germany; 17 Gemeinschaftspraxis für Humangenetik & Genetische Labore (Peters, Kleier, Preusse), Altonaer Str. 61-63, Hamburg, 20357 Germany; 18 Praxis für Humangenetik Freiburg, Heinrich-von-Stephan-Str. 5, Freiburg, 79100 Germany; 19 Institute of Human Genetics, University Hospital RWTH Aachen, Pauwelsstr. 30, Aachen, 52074 Germany; 20 Institute of Human Genetics, University Hospital Magdeburg, Leipziger Str. 44, Magdeburg, 39120 Germany

**Keywords:** Cancer epidemiology, Paediatric cancer, Cancer epigenetics

## Abstract

**Background:**

Beckwith-Wiedemann syndrome (BWS) is a cancer predisposition syndrome caused by defects on chromosome 11p15.5. The quantitative cancer risks in BWS patients depend on the underlying (epi)genotype but have not yet been assessed in a population-based manner.

**Methods:**

We identified a group of 321 individuals with a molecularly confirmed diagnosis of BWS and analysed the cancer incidence up to age 15 years and cancer spectrum by matching their data with the German Childhood Cancer Registry.

**Results:**

We observed 13 cases of cancer in the entire BWS cohort vs 0.4 expected. This corresponds to a 33-fold increased risk (standardised incidence ratio (SIR) = 32.6; 95% confidence interval = 17.3-55.7). The specific cancers included hepatoblastoma (*n* = 6); nephroblastoma (*n* = 4); astrocytoma (*n* = 1); neuroblastoma (*n* = 1) and adrenocortical carcinoma (*n* = 1). The cancer SIR was highest in patients with a paternal uniparental disomy of 11p15.5 (UPDpat). A high cancer risk remained when cases of cancer diagnosed prior to the BWS diagnosis were excluded.

**Conclusions:**

This study confirms an increased cancer risk in children with BWS. Our findings suggest that the highest cancer risk is associated with UPDpat. We were unable to confirm an excessive cancer risk in patients with IC1 gain of methylation (IC1-GOM) and this finding requires further investigation.

## Background

Beckwith-Wiedemann Syndrome (BWS; MIM 130650) is a multisystem human imprinting disorder. Characteristic features are macrosomia, macroglossia, visceromegaly, abdominal wall defects, neonatal hypoglycaemia and an increased occurrence of embryonal tumours.^1^ The prevalence is estimated to be 1:10,000 live births.^2^ BWS is caused by genetic or epigenetic defects of the Imprinting Centers (IC) in the chromosome 11p15.5 region. The IC1 (*H19/IGF2*:IG DMR) regulates the expression of the *H19* and *IGF2* genes, while the expression of *CDKN1C*, *KCNQ10T1* and *KCNQ1* is under the control of IC2 (*KCNQ1OT1*:TSS DMR).^1^ The term Beckwith-Wiedemann spectrum (BWSp) describes “classical BWS without a molecular diagnosis and BWS-related phenotypes with an 11p15.5 molecular anomaly”.^1^

The molecular BWSp subgroups are: (a) *KCNQ1OT1*:TSS DMR (IC2) loss of methylation (IC2-LOM); (b) chromosome 11p15.5 paternal uniparental disomy (UPDpat); (c) *H19/IGF2*:IG DMR (IC1) gain of methylation (IC1-GOM); (d) pathogenetic variants in *CDKN1C* and (e) deletions/duplications of 11p15.5.^3^

The previously reported cancer incidences in BWSp children range from 2.5 to 28.6%.^4–6^ Different authors described an (epi)genotype–phenotype correlation and reported that BWSp patients with IC1-GOM and UPDpat have the highest cancer risk.^7^ The most common cancers associated with BWSp are nephroblastoma and hepatoblastoma.^4^ More rare cancers described in children with BWSp include acute lymphoblastic leukaemia,^8,9^ adrenocortical carcinoma,^10^ hemangiotheloma,^11^ melanoma,^9,12^ neuroblastoma and ganglioneuroblastoma,^9^ pancreatoblastoma,^13^ phaeochromocytoma,^14^ rhabdomyosarcoma^15^ and thyroid cancer.^16^ The published overall childhood cancer risk in the various subgroups are the following: IC2-LOM 2.6%, IC1-GOM 28.1% (mainly Wilms tumour), UPDpat 16% (mainly Wilms tumour and hepatoblastoma), *CDKN1C* pathogenic variant 6.9% (mainly neuroblastoma) (reviewed in^1^). Substantially higher overall cancer risks were found in a recent cohort study (IC2-LOM: 4.4%; IC1-GOM: 51.6%; UPDpat: 29.7%).^17^

However, the cancer risk in BWSp with various subtypes has not yet been quantified in a population-based manner. Therefore, we investigated the occurrence of childhood cancer among individuals from Germany with a genetically confirmed diagnosis of BWSp diagnosed between 2006 and 2018 by matching their data with the German Childhood Cancer Registry (GCCR).^18^

## Methods

We performed a systematic survey among molecular genetic laboratories in Germany that offer BWSp testing. Nineteen different institutions were identified, of which 17 provided their data. One communicated that they had no molecularly confirmed cases. Another one did not respond. Generally, genetic testing was performed by methylation-specific multiplex ligation-dependent probe amplification (MS MLPA; Kit ME030, MRC Holland, Amsterdam, The Netherlands), and this assay includes both copy number and methylation analysis. Thus, duplications and UPD can be discriminated. A total of 321 individuals with molecularly confirmed BWSp were identified. We investigated the occurrence of childhood cancer in this cohort by matching their data with the GCCR.^18^ We also included an analysis of both benign and malignant tumours of the central nervous system (the GCCR only collects benign tumours from the CNS, but not from other regions). The study was approved by the ethical review committee at the Hannover Medical School (#3281-2016). The matching procedure has been described elsewhere.^19^ Briefly, molecularly defined cases of BWSp confirmed between 1 January 2006 and 31 December 2018 were identified at 17 main German laboratories that offer molecular genetic testing for BWSp. Using names and dates of birth, we matched laboratory-diagnosed cases of BWSp with the database of the GCCR (59,205 childhood cancer patients at cut-off date). As described previously, the GCCR registers ~97% of all German childhood malignancies diagnosed at an age of up to 15 years in Germany since 1980.^18^ All diagnoses defined in the International Classification of Childhood Cancer, Third edition are registered systematically.^20^

The personal identifiers from individuals confirmed as having BWSp by the laboratories were encrypted using the same asymmetric key that is employed by GCCR. A matching procedure identified individuals with BWSp and childhood cancer, as described previously.^19,21^ We accumulated person-years of observation from birth to date of last follow-up, as described previously.^19,21^ Person-years of observation were left censored before 1 January 1980 and were observed through 31 December 2018, or right censored at cancer diagnosis, or their 15th birthday, or their death, if recorded, whichever occurred first. Vital status information in the laboratory data was incomplete; individuals without a specified status were assumed to be alive at the cut-off by default. All cancer cases younger than 15 years from the registry with an encrypted name between 1980 and 2018 were included in the matching procedure. Comparisons are presented as standardised incidence ratios (SIRs) with an exact 95% confidence interval (CI), as described previously.^19,21^ Expected values were derived from the same subset of the GCCR data, which was used for the matching procedure.^18^ SIRs were compared as described elsewhere.^22^

## Results

We identified 321 individuals with a molecularly confirmed diagnosis of BWSp whose childhood period overlapped with the GCCR activity. Laboratory tests were conducted between 2006 and 2018. The following epigenetic and genetic defects were identified in peripheral blood: IC2-LOM (*n* = 208), UPDpat (*n* = 64), IC1-GOM (*n* = 31), duplication/deletion (*n* = 12), pathogenetic variant in *CDKN1C* (*n* = 6). This distribution is similar to the distribution known from other BWSp cohorts.^5–7^

The 321 BWSp patients included in the final analytic data file contributed 2306.6 person-years of observation. Birth years ranged from 1978 to 2018. Age at genetic testing ranged from 0 to 38 years. The male-to-female ratio was 0.84. Thirteen patients with cancer, presenting between 1989 and 2018 and diagnosed with a BWSp-causing molecular defect between the years 2006 and 2018 were identified in the BWSp cohort (Table 1).

**Table 1:** Description of the 13 individuals with BWSp who developed cancer.

Patient	Sex	Age (months) at genetic testing	Epigenetic mutation	Neoplasm (age in months)
1	M	6	IC2-LOM	Astrocytoma (45)
2^a^	F	0^b^	UPDpat	Neuroblastoma & Ganglioneuroblastoma (0^b^)
3^a^	F	40	UPDpat	Hepatoblastoma (5)
4^a^	M	14	UPDpat	Nephroblastoma (12)
5^a^	F	143	UPDpat	Adrenocortical carcinoma (10)
6^a^	M	63	UPDpat	Hepatoblastoma (3)
7	F	3	IC2-LOM	Hepatoblastoma (12)
8^a^	F	6	IC2-LOM	Nephroblastoma (3)
9^a^	M	349 (29 y)	UPDpat	Nephroblastoma (41)
10^a^	M	277 (23 y)	UPDpat	Hepatoblastoma (10)
11	M	3	UPDpat	Hepatoblastoma (7)
12	F	7	UPDpat	Nephroblastoma (7)
13^a^	M	1	UPDpat	Hepatoblastoma (0)

*F* female, *M* male, *IC2-LOM* IC2 loss of methylation, *UPDpat* paternal uniparental disomy of 11p15.5, *y* years.

^a^Excluded cases for sensitivity analysis (see results): In these patients the positive cancer history may have prompted molecular testing for BWSp.

^b^The newborn had the tumour diagnosis a few days before the BWSp diagnosis.

On the basis of all person-years and the age distribution of the studied population, 0.4 cases of childhood cancer, all sites combined, would be expected vs 13 observed, a 33-fold increase (SIR = 32.6; 95% CI = 17.3–55.7) (Table 2). Age at diagnosis ranged from 0 to 3.8 years. The childhood cancer risk in BWSp patients due to UPDpat was 153-fold increased (SIR = 152.5, 95% CI = 73.1–280.5), whereas patients with IC2-LOM had an 11-fold (SIR = 11.4, 95% CI = 2.3–33.2) increased risk. There were no cancers observed either among the 31 IC1-GOM patients (268.9 PY; 0.0449 cases expected), the 12 duplication/deletion patients or the six patients with a pathogenetic variant in *CDKN1C*. The cancer risk up to the 15th birthday was 4.4% in the entire cohort, 17.6% for patients with UPDpat and 1.6% for patients with IC2-LOM.

**Table 2:** Molecular subgroup-dependent categorisation of BWSp patients identified in the 17 participating laboratories.

		Cases of cancer^a^				
Molecular diagnosis	*N*	Observed	Expected	PY	SIR, 95% CI	Cancer risk up to 15th birthday (%)
Sum	321	13	0.3991	2306.6	32.6 (17.3–55.7)	4.4
IC2-LOM	208	3	0.2639	1515.9	11.4 (2.3–33.2)	1.6
UPDpat	64	10	0.0656	361.4	152.5 (73.1–280.5)	17.6
IC1-GOM	31	0	0.0449	268.9	0.0 (0.0–82.2)	0.0
Dup/Del	12	0	0.0168	106.7	0.0 (0.0–219.9)	0.0
CDKN1C	6	0	0.0079	53.6	0.0 (0.0–465.7)	0.0

*CI* confidence interval, *Dup* duplication, *Del* deletion, *IC* imprinting center, *IC1-GOM* IC1 gain of methylation, *IC2-LOM* IC2 loss of methylation, *PY* person-years, *SIR* standardised incidence ratio, *UPDpat* paternal uniparental disomy of 11p15.5.

^a^Data from the German Childhood Cancer Registry (see Materials and Methods for details).

SIRs of selected cancers in individuals with BWSp by cancer type are given in Table 3. High SIRs were observed for hepatoblastoma (UPDpat: SIR = 3128.7, 95% CI = 1015.9–7301.2; IC2-LOM: SIR = 174.1, 95% CI = 4.4–970.1), nephroblastoma (UPDpat: SIR = 575.1, 95% CI = 118.6–1680.5; IC2-LOM: SIR = 51.5, 95% CI = 1.30–287.0) and adrenocortical carcinoma (UPDpat: SIR = 10639.9, 95% CI = 269.4–59281.7).

**Table 3:** Standardised incidence ratios for specific cancers in patients with BWSp with UPDpat and IC2-LOM.

			Cases of cancer^a^			
Molecular diagnosis	Cancer type	*N*	Observed	Expected	PY	SIR, 95% CI
UPDpat	Hepatoblastoma	64	5	0.0016	361.4	3128.7 (1015.9–7301.2)
	Nephroblastoma		3	0.0052	361.4	575.1 (118.6–1680.5)
	Neuroblastoma^b^		1	0.0077	361.4	129.0 (3.3–719.0)
	ACC		1	0.0001	361.4	10639.9 (269.4–59281.7)
IC2-LOM	Astrocytoma	208	1	0.0254	1515.9	39.3 (1.0–219.7)
	Hepatoblastoma		1	0.0057	1515.9	174.1 (4.4–970.1)
	Nephroblastoma		1	0.0194	1515.9	51.5 (1.30–287.0)

*ACC* adrenocortical carcinoma, *CI* confidence interval, *IC2-LOM* IC2 loss of methylation, *PY* person-years, *SIR* standardised incidence ratio, *UPDpat* paternal uniparental disomy of 11p15.5.1.

^a^Data from the German Childhood Cancer Registry (see Materials and Methods for details).

^b^The patient had neuroblastoma and ganglioneuroblastoma.

To reduce selection bias, we conducted a sensitivity analysis by excluding the nine cases in whom the cancer diagnosis was made prior to the molecular BWSp (excluded cases are marked in Table 1). After exclusion of these cases, we calculated an unbiased 12-fold increased cancer risk (SIR = 12.3, 95% CI = 3.4–31.5) for all BWSp patients combined.

## Discussion

This is, to our knowledge, the first population-based study to quantify the cancer risk in children with BWSp with IC1-LOM, IC2-GOM, UPDpat, deletions/duplications and pathogenetic variants in *CDKN1C*. We observed a significant excess risk for all childhood cancers combined compared with the general population. The elevated overall cancer risk was primarily due to significant excesses of hepatoblastoma and nephroblastoma. Notably, we also observed rare cancers, such as astrocytoma and ganglioneuroblastoma. We found one previous case of ganglioneuroblastoma^9^ and another case of brain glioma^23^ in the literature.

The chromosome 11p15.5 region that is altered in individuals with BWSp harbours several imprinted genes, among them *CDKN1C*, *IGF2* and *H19*, which are regulators of growth.^24^ Therefore, the observed high cancer risk in individuals with BWSp who carry a constitutional or mosaic lesion in the 11p15.5 region is biologically plausible. Although a number of studies have shown a significant association between cancer risk and BWSp molecular subtype,^6^ a population-based epidemiologic study has not yet been conducted to address this topic quantitatively. This approach has the advantage of being less prone to ascertainment bias and allows for the calculation of SIRs. Thus, methodological differences in study design, including different ascertainment and follow-up criteria, prevent a direct comparison of these studies with ours. Brioude et al. studied 407 patients with BWSp including 257 patients with IC2-LOM, 81 with UPDpat, 35 with IC1-GOM and 34 individuals with a pathogenic variant in *CDKN1C*.^6^ Patients were either followed at the authors’ centre or referred by other centres for suspected BWSp. A form was used to collect data at the time of diagnosis and at follow-up. 8.6% of all patients developed tumours. The highest cancer incidence was observed among BWSp children with IC1-GOM (28.6%) and UPDpat (17.3%).

Maas et al. performed a literature review evaluating reported data from 1971 BWSp patients.^5^ 155/1923 (8%) of BWSp children developed tumours (IC2-LOM 2.6% with cancer, IC1-GOM 28%, UPDpat 16% and pathogenetic variants in *CDKN1C* 6.9%).

Cooper et al. studied additional 200 individuals with a confirmed molecular genetic diagnosis of BWSp.^7^ Cancer was more frequent in individuals with IC1-GOM or UPDpat (13%) than in those with a pathogenetic variant in *CDKN1C* or IC2-LOM (1%).

Duffy et al. described a cohort of 344 individuals with BWSp registered in the growth and genetic/epigenetic disorder registry at Children’s Hospital of Philadelphia. Two hundred and nineteen individuals were included in the subgroup tumour analysis. A tumour was diagnosed in 43/219 (19.6%) of individuals: IC2-LOM: 5/114 (4.4%), IC1-GOM: 16/31 (51.6%) and UPDpat: 22/74 (29.7%).^17^

In contrast to these published studies, we have collected molecularly confirmed cases of BWSp in a population-based manner and have calculated the relative cancer risks compared to population-based incidence rates in an effort to analyse the cancer risks employing a less biased method.

The overall cumulative childhood cancer incidence observed in this cohort are in agreement with the cancer incidences described in previous studies.^5–7^ We were able to demonstrate the elevated childhood cancer risk in individuals with BWSp compared to the population average risk, even when all patients with a cancer diagnosis prior to the BWSp diagnosis were excluded. In agreement with previous studies, we found a high cancer risk to be associated with UPDpat.^1^ The risk in patients with UPDpat was significantly higher than the risk in IC2-LOM (Table 2). To our surprise, we observed no cases of cancer in BWSp children with IC1-GOM; however, this finding was not statistically significant. This molecular subgroup is believed to be associated with the highest cancer risk.^1^ Larger cohorts of patients with IC1-GOM may be required to calculate the cancer risk more precisely. In agreement with previous reports, we observed a cumulative childhood cancer risk of <3% in BWSp children with IC2-LOM.^1,5–7^ Notably, even with small percentage risk this was still an 11-fold increased cancer risk with a wide CI. Although some molecular subgroups have a lower risk of cancer, parents who decline surveillance^1^ should be aware that the absolute of cancer is still increased in these groups compared with the general population.

Our study has several limitations. (1) The study is not powered to detect any increase (or decrease) in incidence of the more common childhood cancers. Combining data with other cohorts may be a strategy to reach this goal. (2) We were unable to ascertain cancers in patients after their 15th birthday, as the case-identifying resource was a childhood cancer registry. Germany does not yet have an equivalent national cancer registry for adults. Thus, the risk of adolescent or adult-onset cancers in BWSp cannot be investigated here. Notably, new evidence does support an increase in cancer risk for BWSp beyond childhood.^25^ (3) We did not have access to patient medical records, and thus could not determine each subject’s age or date at clinical syndrome diagnosis in case this age differed from the molecular diagnosis. It is likely that, in some instances, the BWSp diagnosis was prompted by the development of a childhood cancer. Of note, in nine patients, the cancer was diagnosed before the molecular confirmation of BWSp (Table 1). If we exclude these nine cases from our analysis, the remaining cancer risk for all cancers among all BWSp individuals combined remains significantly elevated (SIR = 12.3, 95% CI = 3.4–31.5). Conversely, patients with BWSp are more likely to undergo cancer surveillance potentially leading to an overestimation of certain cancer diagnoses. (4) Vital status information in the laboratory data was incomplete; individuals without a specified status were assumed to be alive at the cut-off by default potentially leading to an underestimation of the cancer risk. (5) As a consequence of identifying susceptible individuals through genetic diagnostic labs, our analytic cohort excludes BWSp patients who have never undergone genetic testing (i.e. who were diagnosed clinically without molecular confirmation) as well as BWSp patients who had a clinical diagnosis but negative molecular testing. There is no way to evaluate the impact of this subgroup’s absence on our analysis. Given the fact that the percentage of tested and untested individuals with BWSp is unknown, we are unable to calculate the BWSp incidence based on our data. (6) The lack of replicating previous results on an increased cancer risk for IC1-GOM might reflect the smaller number of Person-Years analysed in this group (5-fold less than for IC2-LOM). (7) One German laboratory with molecularly confirmed cases of BWSp did not participate in this research and it cannot be ruled out that some additional patients received their molecular tests in laboratories that we did not identify, because their diagnostic portfolio is not publicly available, or in laboratories from other countries. However, outsourcing of genetic testing is unusual in Germany, as health insurance companies routinely cover genetic testing only if done in a national lab.

In conclusion, this population-based study demonstrated an increased childhood cancer risk, especially hepatoblastoma and nephroblastoma, in children with BWSp. Our findings suggest that in children with BWSp the highest cancer risk is associated with UPDpat, whereas the risk in IC1-GOM requires further investigation. Our data are consistent with current surveillance guidelines.^1^

## Data Availability

Data are held within the Department of Pediatric Hematology and Oncology at Hannover Medical School and are available on application.
